# Exploring Pulmonary Dysfunction in Parkinson’s Disease: The Role of Impulse Oscillometry—A Systematic Review

**DOI:** 10.3390/jcm14113730

**Published:** 2025-05-26

**Authors:** Alexandra-Cristiana Gache, Elena Danteș, Elena Mocanu, Andreea-Cristina Postu, Cristian Opariuc-Dan, Any Axelerad

**Affiliations:** 1 Department of Pneumology, Faculty of Medicine, Campus—Corp B, Ovidius University of Constanta, 1 University Alley, 900470 Constanta, Romania; alexandra.belu@365.univ-ovidius.ro (A.-C.G.); elena.dantes@365.univ-ovidius.ro (E.D.); 2 Medical Doctoral School, Faculty of Medicine, Campus—Corp B, Ovidius University of Constanta, 1 University Alley, 900470 Constanta, Romania; docuaxi@yahoo.com; 3 Clinical Hospital of Pneumopthisiology Constanta, 40 Santinelei Street, 900002 Constanta, Romania; postu.andreea@gmail.com; 4 Department of Public Health and Management, Faculty of Medicine, Campus—Corp B, Ovidius University of Constanta, 1 University Alley, 900470 Constanta, Romania; 5 Department of Administrative Sciences, Faculty of Law and Administrative Sciences, Campus—Corp A, Ovidius University of Constanta, 1 University Alley, 900470 Constanta, Romania; cristian.opariuc@365.univ-ovidius.ro; 6 Department of Neurology, Faculty of Medicine, Campus—Corp B, Ovidius University of Constanta, 1 University Alley, 900470 Constanta, Romania

**Keywords:** Parkinson’s disease, impulse oscillometry, impedance, spirometry, pulmonary function, neurodegenerative diseases

## Abstract

**Background/Objectives**: This systematic review aimed to synthesize current evidence on the use of impulse oscillometry (IOS) in assessing pulmonary function in patients with Parkinson’s disease (PD). IOS, as an effort-independent method, may offer advantages over conventional spirometry in detecting early or subclinical respiratory impairment in neurologically compromised populations. **Methods**: A systematic search was conducted across PubMed, Web of Science, Scopus, ScienceDirect and Google Scholar for observational studies published up to March 2025. The included studies involved patients diagnosed with PD who underwent respiratory assessment using IOS, either alone or in combination with spirometry. Data on IOS parameters (R5, R20, X5, AX) and their associations with disease severity, spirometric values or autonomic markers were extracted and analyzed qualitatively. **Results**: Four studies, published between 2020 and 2023, met the inclusion criteria. IOS revealed increased airway resistance in early-stage PD and inverse correlations with spirometric indices such as FEV1 and PEF. One study demonstrated significant correlations between IOS parameters and parasympathetic heart rate variable indices, suggesting autonomic involvement. IOS also showed stability across dopaminergic treatment states, highlighting its reliability in longitudinal monitoring. **Conclusions**: IOS appears to be a promising adjunct to traditional respiratory assessment in PD, capable of identifying subtle mechanical and autonomic dysfunctions. Despite encouraging results, the current evidence remains limited and further large-scale, longitudinal studies are needed to validate its clinical utility.

## 1. Introduction

Parkinson’s disease (PD) is a gradual neurological disorder, associated with the progressive degeneration of dopaminergic neurons in the substantia nigra. Typically manifesting in later life, PD is characterized primarily by bradykinesia (generalized slowness of movement), accompanied by at least one additional cardinal motor feature, like resting tremor or muscular rigidity [1,2].

The global burden of PD has increased markedly over recent decades. Its prevalence has more than doubled in the past 25 years, with estimates from 2019 indicating that over 8.5 million individuals were living with the condition. In the same year, PD was responsible for approximately 5.8 million disability-adjusted life years (DALYs), representing an 81% increase since 2000, and accounted for 329.000 deaths worldwide [3].

Non-motor symptoms are now recognized as key features of the prodromal disease stages. They include neuropsychiatric symptoms (depression, anxiety), autonomic dysfunction (constipation, orthostatic hypotension), hyposmia and a range of sleep-related disturbances such as rapid eye movement (REM) sleep behavior disorder [4]. Unfortunately, they are frequently underdiagnosed and become increasingly challenging to manage as the disease progresses [5].

Multiple converging mechanisms contribute to the pathogenesis of PD beyond the classical degeneration of dopaminergic neurons in the substantia nigra. Chronic neuroinflammation, sustained microglial activation and oxidative stress play central roles in driving neuronal vulnerability and α-synuclein aggregation. Experimental models implicate microglial NADPH oxidase in dopaminergic cell death via superoxide radical generation, while other glial components—such as tumor necrosis factor-α and Fc gamma receptors—amplify neuroinflammatory cascades. Although nitric oxide has been associated with neurotoxicity in rodent models, human microglia produce minimal NO, suggesting species-specific differences [6].

In parallel, disruption of neuropeptidergic systems, particularly the orexinergic pathway, may further exacerbate neurodegeneration. Orexin, a hypothalamic neuropeptide involved in arousal and metabolic regulation, is produced by neurons that are selectively affected in Lewy body diseases such as Parkinson’s disease and dementia with Lewy bodies. Degeneration of orexin-producing neurons, along with damage to the tuberomammillary and lateral tuberal nuclei, have been documented in PD. Moreover, altered orexin levels have been linked to disease severity, supporting a broader role for hypothalamic dysfunction in both motor and non-motor symptomatology. Collectively, these mechanisms underscore the complex, multisystemic nature of PD and its potential impact on respiratory and autonomic regulation [7].

Respiratory involvement in PD has long been present, as suggested by early clinical observations, including James Parkinson’s description of a patient who “fetched his breath rather hard” [8]. Despite this early insight, respiratory abnormalities have remained relatively understudied compared to the cardinal motor features of the disease. These disturbances are now understood to include a range of manifestations—for example, upper airway obstruction, restrictive ventilatory patterns, impaired respiratory muscle function and central ventilatory dysregulation—which can significantly contribute to morbidity and mortality in PD [9,10].

Although spirometry has proven effective in detecting respiratory abnormalities and have consistently shown measurable ventilatory dysfunctions in patients with PD, demonstrated by significant reductions in FEV_1_, FVC and PEFR, it remains an effort-dependent technique. However, its clinical utility is often limited by patients’ motor impairments [11,12]. These limitations underscore the need for alternative methods like impulse oscillometry (IOS), which allows the passive assessment of respiratory mechanics and may offer greater sensitivity in detecting subtle or early dysfunctions in this population [13].

IOS is a non-invasive, effort-independent method that measures airway resistance and reactance during normal breathing. It is sensitive to small airway changes and suitable for patients who cannot perform spirometry reliably [14]. IOS delivers a brief pressure impulse containing multiple frequencies (5–30 Hz) to assess lung mechanics during tidal breathing and provides regional information on airway function, with low frequencies assessing peripheral airways and high frequencies reflecting central airways [14]. Key parameters include R5 (resistance at 5 Hz), which quantifies the total airway resistance encompassing both central and peripheral airways, and R20 (resistance at 20 Hz), which primarily characterizes central airway resistance. The difference R5–R20 serves as a marker of peripheral airway dysfunction by capturing the frequency dependence of resistance. X5 (reactance at 5 Hz) characterizes the combined elastic and inertial properties of the lungs, with more negative values indicating reduced compliance or increased stiffness. AX (area under the reactance curve) summarizes the cumulative elastic load imposed by the lung periphery, while Fres (resonant frequency)—the point at which reactance crosses zero—typically increases in both obstructive and restrictive pathologies. Together, these parameters offer a nuanced, non-effort-dependent assessment of airway mechanics and are particularly valuable for detecting early or subclinical dysfunction in conditions involving both central and peripheral airway pathology [14,15].

Despite its theoretical and clinical advantages, the use of IOS in PD remains limited and insufficiently studied. To date, no systematic review has synthesized this evidence, and the diagnostic or prognostic relevance of IOS in this population remains unclear.

This review aims to evaluate the current literature on IOS in PD, clarify its potential clinical utility and highlight gaps for future research.

## 2. Materials and Methods

This systematic literature review was conducted in accordance with the Preferred Reporting Items for Systematic Reviews and Meta-Analyses (PRISMA) guidelines, an internationally recognized and widely adopted methodological framework [16]. The review was prospectively registered in the PROSPERO database (International Prospective Register of Systematic Reviews) under the registration number CRD420251021426 (31 March 2025).

### 2.1. Search Strategy

In order to identify eligible studies, an extensive search was conducted in the following electronic databases: PubMed, Web of Science, Scopus, ScienceDirect (Elsevier) and other platforms—Google Scholar, using the following Boolean expression: (“Parkinson” AND “oscillometry” AND “pulmonary function”) OR (“Parkinson” AND “impedance” AND “lung function”) (Table 1).

The research question guiding this review was structured according to the Patient/Problem, Intervention, Comparison and Outcome framework (PICO), as follows: In patients with Parkinson’s disease (P), how does pulmonary assessment using impulse oscillometry (I) compare to spirometry (C) in detecting early respiratory dysfunction and its clinical relevance (O)?

### 2.2. Eligibility Criteria

Articles were selected based on the following inclusion criteria: studies published in English, available as free full text or closed-access articles obtained directly from the authors, involving patients with a confirmed diagnosis of PD who underwent respiratory function assessment using IOS, either as a standalone or in combination with conventional pulmonary function testing. Eligible publications were required to fall under the category of evidence-based medicine (e.g., observational studies with clearly defined methodologies) and were considered regardless of PD stage or comorbid conditions, provided that oscillometric methods were employed in the evaluation of pulmonary function. The following filters were applied in PubMed: free full text; publication types limited to clinical and randomized controlled trials. Exclusion criteria were based on editorials, expert opinions and narrative reviews without a structured methodology were also excluded.

### 2.3. Data Extraction

Citations retrieved from PubMed, ScienceDirect (Elsevier), Web of Science, Scopus and Google Scholar were imported into Zotero for management. Duplicates were identified and removed after exporting the library to Microsoft Excel, using the “remove duplicates” function.

### 2.4. Risk of Bias

The risk of bias for the included studies was assessed using the ROBINS-I V2 (Risk of Bias in Non-randomized Studies of Interventions, Version 2) tool, which is specifically designed to evaluate non-randomized observational studies. This instrument enables the appraisal of material bias across seven key domains: bias due to confounding, bias in classification of interventions, bias in selection of participants into the study, bias due to deviations from intended interventions, bias due to missing data, bias in measurement of outcomes and bias in selection of the reported result.

The assessment was performed by two reviewers (E.D, A.A.), with any discrepancies resolved through consensus. This rigorous approach ensured a transparent and comprehensive evaluation of internal validity across the studies included in this review.

## 3. Results

### 3.1. Search

A total of 201 records were identified through systematic database searches conducted across five major platforms. Following the removal of 86 duplicate records, 115 unique citations were retained for preliminary screening. Titles and abstracts were independently screened by two reviewers (A.-C.G, E.M.) to assess their relevance with respect to the predefined eligibility criteria. As a result, 102 records were excluded due to their lack of alignment with the central research question, absence of relevant methodology or focus on unrelated populations or diagnostic techniques.

Subsequently, 13 articles were considered for further consideration. A second round of screening by two additional reviewers (A.-C.P., C.O.-D.), based on a more refined assessment of abstracts, led to the exclusion of 7 additional records, with either a lack of methodological clarity, failure to report primary pulmonary function outcomes or were deemed insufficiently focused on PD.

The remaining six articles were reviewed in depth by all authors (A.-C.G., A.-C.P., E.D., E.M., C.O.-D., A.A.) to verify their alignment with the predefined inclusion parameters. Of these, two were excluded: one article could not be retrieved in full text despite repeated attempts to access it through institutional channels and scientific correspondence, while the other was a narrative review. Although the latter did not qualify for inclusion in the evidence synthesis due to its non-empirical nature, it provided valuable conceptual support for the potential role of impulse oscillometry in evaluating respiratory mechanics among patients with neuromuscular and chest wall disorders [17].

**Figure 1:**
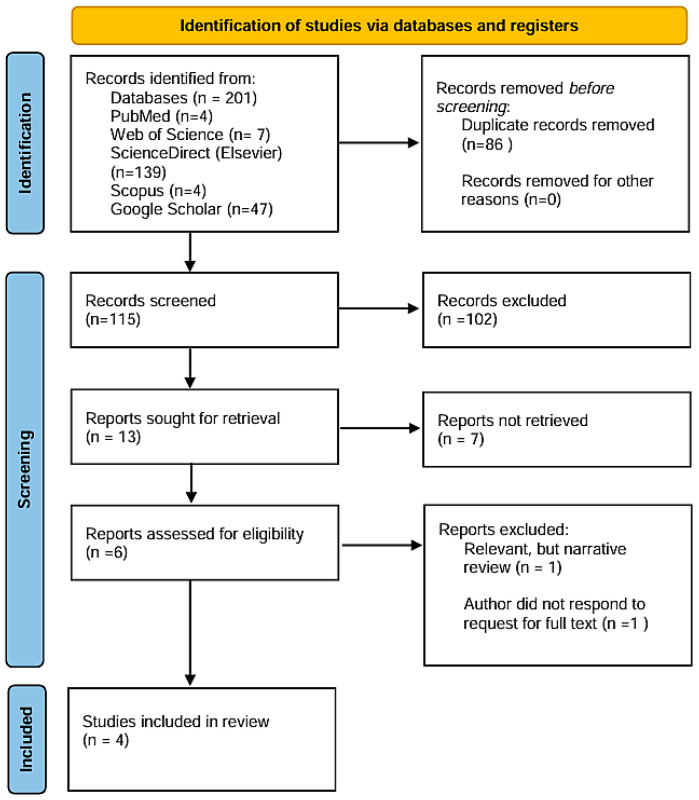
Our adapted PRISMA flow diagram outlines the process of study identification, screening, eligibility assessment and inclusion.

A total of four studies met the eligibility criteria and are presented in Table 2, which includes information on authorship, year of publication, study design, PD’s stage—Hoehn and Yahr Classification (H&Y), methods used for respiratory assessment and main findings. The included research primarily comprised observational studies evaluating pulmonary function in individuals with PD using IOS, either independently or in conjunction with conventional spirometry (Figure 1).

### 3.2. Description of Selected Studies

The four studies in this systematic review were published between 2020 and 2023 and investigated respiratory function in patients with PD using IOS, either alone or in conjunction with conventional spirometry. All studies employed observational designs and involved adult PD populations across mild to moderate disease stages, using the H&Y scale. Sample sizes ranged from 20 to 67 participants; half of the included studies incorporated a control group.

Caldas et al. (2023) conducted the most detailed and methodologically refined investigation, stratifying patients by disease severity and smoking status [13]. Their analysis demonstrated progressive increases in peripheral resistance (Rp) and Fres with advancing disease stage. Moreover, the diagnostic performance of IOS was notable: Rp exhibited strong discriminative ability in early-stage PD (AUC = 0.858), while Fres proved highly sensitive in more advanced stages (AUC = 0.948). Importantly, IOS was able to distinguish subtle pathophysiological changes even in patients with normal spirometric indices, supporting its potential utility as a sensitive tool for the early detection of respiratory involvement in PD. The study also highlighted the impact of smoking on IOS parameters, particularly in exacerbating central and peripheral resistance, suggesting the need for careful consideration of smoking status in pulmonary assessments [13].

A subsequent study conducted by Oliviera et al. in 2022 evaluated respiratory function in a group of 21 individuals with a mild-stage PD using both IOS and spirometry under “on” and “off” phases of levodopa treatment [18]. Unlike spirometry, which revealed early obstructive tendencies (e.g., reduced FEV1 and peak expiratory flow), IOS parameters remained within reference limits across both pharmacological states. The observed differences between medication phases were minimal in IOS measures (η^2^ = 0.043), reinforcing the notion that IOS may provide a stable, effort-independent assessment of respiratory function, relatively unaffected by dopaminergic fluctuations. The authors suggest that while IOS may be less responsive to short-term medication effects, it may serve as a reliable baseline tool in longitudinal respiratory monitoring. Moreover, the study illustrates the potential for discordance between effort-dependent and passive measurements in the early clinical course of PD [18].

Sampath and colleagues published two complementary studies that investigated respiratory involvement in Parkinson’s disease from distinct perspectives, using IOS as the principal assessment method. The first study, published in 2020, included 30 patients and aimed to detect early alterations in pulmonary function through IOS. The results showed significantly elevated resistance at R5 and R20 values in stage II patients compared to those in stage I, indicating increased resistance in both total and central airways. Additionally, R20 was inversely correlated with spirometric parameters such as FEV_1_ and PEF, suggesting that IOS can identify subtle mechanical changes that may not be captured by conventional spirometry.

In their 2022 study, the same authors explored the relationship between IOS parameters and HRV analysis to explore the interplay between respiratory impedance and autonomic dysfunction in PD. The study included 30 patients diagnosed with PD (25 retained for analysis after artifact removal). The investigation revealed that central airway resistance (R20) was significantly correlated with parasympathetic HRV indices, such as root mean square of successive differences and high-frequency power (HF). These associations suggest that IOS may detect subtle alterations in airway tone and compliance related to autonomic dysregulation. The study provides novel evidence that respiratory impedance—particularly in central airways—may reflect extrapyramidal autonomic involvement in PD, offering a potential non-invasive marker of dysautonomia in conjunction with pulmonary assessment [19,20].

### 3.3. Risk of Bias Assessment

**Figure 2:**
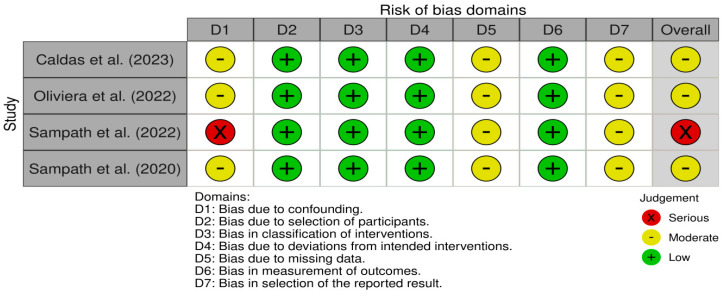
Risk of bias across individual studies [13,18,19,20].

An overall risk of bias judgment was generated for each study based on the highest level of bias identified in any domain. Judgments were categorized as low, moderate, serious or critical risk of bias (Figure 2).

Most included studies were at moderate risk of bias, with only one study (Sampath et al., 2022 [19]) showing a serious risk. The most frequent concerns arise from confounding and missing data, which are challenges in observational designs.

Caldas et al. (2023) showed a moderate risk of bias overall [13]. Moderate concerns were mainly due to potential residual confounding and the incomplete reporting of selection processes. Domains such as classification of interventions, deviations from intended interventions and measurement of outcomes were judged at low risk.

Oliviera et al. (2022) was similarly rated at moderate risk [18]. While intervention classification and deviations were well-handled, moderate risks were identified in domains related to confounding, missing data and selective reporting, primarily due to lack of prespecified analysis plans.

Both studies by Sampath et al. exhibited limitations in risk of bias, though differing in severity [19,20]. In 2022, they demonstrated a serious overall risk of bias, primarily driven by inadequate control for confounding factors, which critically affected the interval validity despite low risk ratings across other domains. In contrast, in 2020, they showed a moderate risk of bias, mainly due to concerns regarding the incomplete handling of missing data and potential selective outcome reporting, while maintaining low risk in intervention classification and participant selection. These differences highlight the varying degrees of methodological rigor within the same research group.

## 4. Discussions

Neurodegenerative diseases involve the progressive loss of selectively vulnerable neurons, differing from static neuronal damage caused by toxic or metabolic conditions. These disorders can be classified based on predominant clinical features (e.g., dementia, Parkinsonism), anatomical patterns of degeneration (e.g., extrapyramidal, cerebellar), or underlying molecular abnormalities [21].

### 4.1. Overview of Respiratory Dysfunction in PD

Although PD was first described over two centuries ago, respiratory dysfunction remains one of the most important aspect in disease evolution. These abnormalities often occur in the absence of overt clinical symptoms and are frequently overlooked, despite measurable changes in pulmonary function. The growing recognition of respiratory impairment in PD underscores the need for systematic respiratory strategies aimed to improve patients’ quality of life and overall prognosis [22].

Diagnostic evaluations are commonly guided by general pulmonary function testing principles, with spirometry serving as the most frequently used tool. According to the American Thoracic Society (ATS) and European Respiratory Society (ERS) guidelines, restrictive or obstructive patterns are determined by reductions in FVC and FEV_1_/FVC ratios, respective FEV_1_ and FEV_1_/FVC [23,24].

Restrictive ventilatory patterns are frequently observed in patients with PD with reported prevalence ranging from 28% to 85% [9]. Typically, dyspnea begins as exertional and may progress to dyspnea at rest, often coinciding with worsening motor symptoms such as gait freezing or falls. Even if the underlying pathophysiological mechanisms are not fully elucidated, restrictive respiratory dysfunction is generally attributed to bradykinesia and rigidity of the respiratory muscles, along with reduced thoracic wall compliance [9,25].

Nonetheless, in PD, motor impairments such as rigidity, bradykinesia and tremor may interfere with the execution of effort-dependent maneuvers, potentially leading to underestimation or misclassification of pulmonary dysfunction severity [9,22].

A similar issue is seen in multiple system atrophy (MSA), a related synucleinopathy characterized by widespread autonomic failure and a more aggressive disease course. In contrast to PD, where respiratory dysfunction is often subclinical and primarily influenced by peripheral motor impairments, MSA frequently presents with earlier and more pronounced ventilatory abnormalities. Such features not only contribute to increased morbidity but also complicate respiratory assessment, further emphasizing the need for diagnostic modalities that are less dependent on patient effort [26].

### 4.2. Spirometry and the Challenge of Effort-Dependent Testing

Gartman’s narrative review provides an important theoretical framework for understanding the respiratory assessment landscape in neuromuscular and neurodegenerative diseases. Currently, spirometry remains the primary tool for respiratory evaluation due to its accessibility and its value as a prognostic marker, particularly in diagnosing complications, the need for ventilatory support and survival outcomes [17]. To highlight the role of spirometry in PD, Bogaard and colleagues conducted a study on a cohort of 31 patients, investigating the influence of disease stage on flow-volume loops (FVLs). The results revealed a high number of abnormal FVL patterns, characterized by a combination of upper airway obstructive ventilatory dysfunction and myotonic-like respiratory muscle impairment. These findings underscore the complex interplay between central motor control deficits and peripheral respiratory mechanics in PD and support the utility of spirometric analysis—particularly FVL interpretation—in detecting subclinical respiratory involvement [27].

However, certain neurodegenerative conditions may affect pulmonary volumes depending on the body position during testing, whether seated or supine. For instance, Fromageot et al. notes a significant decrease in vital capacity when spirometry is performed in the supine position in conditions involving diaphragmatic weakness or paralysis, with reported declines ranging from 15% to 25%, or even exceeding 40% in cases of bilateral involvement. Conversely, in other pathologies, vital capacity may paradoxically increase in the supine position, potentially leading to clinical misinterpretation [28].

### 4.3. IOS as an Alternative Tool in PD Respiratory Assessment

Given these limitations, IOS emerges as a valuable alternative method for evaluating pulmonary function in progressive neurological diseases, including PD. Gartman further elaborates on several foundational studies that demonstrates the sensitivity of IOS in detecting mechanical alterations in the respiratory system. Notably, he references the experimental work by van Noord et al. (1986), in which the external restriction of the rib cage and abdomen in healthy individuals produced measurable changes in total respiratory resistance and reactance, as assessed by IOS [29]. These findings demonstrate that IOS can detect variations in chest wall mechanics independent of effort, supporting its application in populations with neuromuscular or extrapyramidal disorders [17,29].

IOS is performed during quiet tidal breathing and does not require forceful respiratory maneuvers, making it especially suitable for individuals with bradykinesia, rigidity or impaired coordination. Moreover, it provides detailed information about both central and peripheral airway resistance and reactance, capturing pathophysiological changes that may not be evident on conventional spirometry [13,17]. The main characteristics of the parameters obtained through spirometry and impulse oscillometry (IOS) are summarized in Table 3.

### 4.4. Evidence from IOS Studies in PD

Building on these theoretical foundations, the present systematic review synthetizes empirical evidence regarding the application of IOS in patients with PD. These theoretical insights set the stage for the current review, which explores the practical use of IOS in patients with Parkinson’s disease. While methodological heterogeneity exists across study designs, sample sizes and IOS parameters analyzed, several shared themes emerge—most notably, the ability of IOS to detect subtle changes in airway mechanism that may not be captured by conventional spirometry.

One of the most significant contributions to this review stems from the two complementary studies conducted by Sampath and colleagues in 2020 and 2022, both of which explored the diagnostic relevance of IOS in PD, from distinct physiological perspectives. The 2020 study was designed to determine whether IOS could detect early-stages respiratory dysfunction by comparing patients in H&Y stages I and II. Out of the 30 patients initially enrolled, only 14 patients were included in the comparative analysis, with 7 patients in stage I and 7 patients in stage II. The study reported that stage II, patients had significantly increased resistance at R5 and R20—R5 values of 0.46 ± 0.12 kPa/L/s compared to 0.33 ± 0.09 kPa/L/s in stage I (*p* < 0.01) and R20 values of 0.39 ± 0.09 kPa/L/s versus 0.29 ± 0.06 kPa/L/s (*p* < 0.05), in stage II—indicating progressive impairment in total and central airway mechanics.

Moreover, the inverse correlation between R20 and spirometric indices such as FEV1 (r = −0.44, *p* < 0.05) and PEF (r = −0.39, *p* < 0.05), suggested that IOS may detect subclinical abnormalities that remain undetected through conventional pulmonary testing [19].

While the 2020 study focused on mechanical respiratory changes, the 2022 follow-up investigation expanded this framework by examining how dysfunction of the autonomic nervous system may influence ventilatory impairment. In this investigation, the authors hypothesized that respiratory abnormalities in PD may not solely arise from motor or muscular rigidity but could also reflect autonomic dysfunction. Using HRV parameters such as high-frequency (HF) power, the study identified significant positive correlations between these variables and R20. This finding is especially notable, as it suggests that increased central airway resistance may serve as an indirect marker of parasympathetic dysregulation. Given that dysautonomia is a well-established non-motor manifestation of PD, the study by Sampath et al. (2022) positions IOS not only as a tool for mechanical assessment, but also as a non-invasive surrogate for evaluating autonomic respiratory control [19,20].

Together, these two studies form a coherent narrative that reinforces the multifaced utility of IOS in PD. The 2020 data emphasize its sensitivity in detecting early mechanical compromise, offering clinicians a means of identifying at-risk patients even in the absence of overt respiratory symptoms. In contrast, the 2022 findings broaden this perspective by implicating IOS in the functional assessment of non-motor domains, specifically the autonomic nervous system’s influence on airway tone and respiratory resistance. The methodological consistency across studies, including comparable sample sizes and standard IOS parameters (R20 in particular), further strengthens the reliability of these observations. However, certain limitations must be acknowledged, such as the relatively small cohort sizes and the absence of long-term follow-up, which limits the ability to evaluate IOS’s predictive capacity over time. Despite this, the findings from Sampath et al. therefore advance the field by illustrating that IOS is not only technically feasible and clinically applicable, but also capable of capturing the complex, multisystemic nature of respiratory impairment in Parkinson’s disease [19,20].

The study conducted by Oliveira et al. offers a nuanced perspective on respiratory function in patients with early-PD, comparing outcomes between IOS and spirometry in both medicated (“on”) and unmedicated (“off”) states. The study included 41 subjects (21 with PD, 20 controls), well-matched across demographic and anthropometric variables, including age, sex, BMI and chest wall mobility. All PD patients were classified as H&Y stage I to II, with a mean score of 1.5 ± 0.4, and received moderate doses of levodopa therapy.

Although no significant differences were observed in IOS parameters (R5, R20, X5) between PD and control groups—or between “on” and “off” medication phases—spirometry revealed early obstructive ventilatory changes in PD patients, including lower FEV_1_/FVC and PEF ratios. These findings are particularly relevant, as they suggest a potential dissociation between the physiological domains captured by IOS and those detected by effort-dependent testing. The absence of IOS alterations, despite spirometric evidence of airflow limitation, may indicate that IOS is less sensitive to mild proximal airway obstruction in the early disease stages, or alternatively, that these impairments are functional and only manifest during active respiratory maneuvers.

Notably, the effect of dopaminergic therapy on pulmonary parameters was minimal (η^2^ = 0.043 for IOS; η^2^ = 0.197 for spirometry), suggesting that medication may have a limited short-term influence on pulmonary mechanics in early-stage PD. While these findings support the use of IOS as a stable and reproducible tool, they also underscore its potential limitations in detecting mild, medication-sensitive respiratory changes. The study is strengthened by its methodological design—accounting for treatment state and using carefully matched controls—but limited by a modest sample size and reliance on the H&Y scale for disease staging. Importantly, the authors note that respiratory dysfunction in PD may be influenced not only by neurodegenerative processes but also by reduced physical activity and chest wall rigidity, both of which progress with disease severity. As such, longitudinal studies are essential to clarify whether the absence of IOS abnormalities in early PD reflects true physiological preservation or diagnostic insensitivity [18].

The investigation by Caldas et al. (2023) provides the most methodologically comprehensive analysis of pulmonary function in PD using IOS [13]. This study enrolled 67 participants, including PD patients stratified by disease stage (mild and moderate) and smoking status, along with age-matched healthy controls. By comparing IOS parameters across these subgroups, the authors identified a progressive increase in p and Fr in correlation with disease severity, supporting the hypothesis that respiratory system involvement advances alongside motor deterioration. Notably, Rp demonstrated strong discriminative power in early-stage PD (AUC = 0.858), while Fr emerged as a highly sensitive marker in later stages (AUC = 0.948). These findings underline the utility of IOS not only for early detection, but also for disease staging.

An important contribution of this study lies in its attention to confounding factors, such as smoking history, which was shown to significantly amplify IOS abnormalities—particularly in central and peripheral resistance measures. Moreover, the study demonstrated that certain IOS alterations were present even in patients with normal spirometric indices, indicating that IOS may uncover subclinical changes undetectable by conventional tests. While the cross-sectional nature of the study limits its prognostic implications, the rigorous stratification and statistically robust outcomes enhance its clinical relevance. Overall, Caldas et al. strengthen the case for implementing IOS in the routine respiratory monitoring of PD patients, especially for early-stage identification and for individuals at risk of respiratory decline but unable to perform effort-based tests like spirometry [13].

To synthesize the findings across studies and highlight the relationship between clinical respiratory patterns, spirometric outcomes and IOS parameters, we present a comparative table below (Table 4). This overview allows for a clearer understanding of where IOS may provide diagnostic advantages or complementary value in Parkinson’s disease.

### 4.5. Limitations of the Studies

A critical aspect emerging from this review is the moderate to serious risk of bias observed across the included studies, primarily related to confounding, missing data and selective reporting. Several studies did not adequately control for key variables such as smoking status, disease duration, dopaminergic treatment state or comorbid conditions, all of which may influence IOS measurements. Additionally, the exclusion of participants due to poor-quality data—particularly in HRV analyses—introduced a risk of attrition bias and may have affected the representativeness of results [13,18,19,20].

Another limitation concerns the heterogeneity in reporting IOS parameters. While all studies reported resistance measures (R5, R20), fewer provided reactance indices (X5, AX, Fres), which are essential for assessing peripheral airway compliance. Inconsistent reporting of effect sizes and model adjustments further limits the ability to evaluate the robustness of findings [18,19,20].

Furthermore, the cross-sectional design of all studies precludes inferences about the progression of respiratory dysfunction in PD. The absence of longitudinal data prevents us from evaluating whether IOS can predict respiratory decline over time [13,18,19,20].

### 4.6. Implications for Future Research

Despite the increasing recognition of respiratory involvement in PD, there are currently no standardized diagnostic criteria specifically targeting pulmonary dysfunction within this population.

In addition to methodological limitations, the current findings must be interpreted in the context of PD’s clinical heterogeneity. Motor subtypes may influence respiratory involvement through distinct pathophysiological pathways, yet none of the reviewed studies accounted for this variation.

Recent data-driven analyses have highlighted the existence of distinct progression subtypes in PD, which may have significant implications for both clinical management and respiratory assessment. Two primary trajectories have been described: a rapidly progressing subtype, characterized by accelerated deterioration in both motor and non-motor domains and a slowly progressing subtype, marked by a more gradual clinical course and reduced symptom burden. These phenotypes were identified through longitudinal modeling across multiple PD cohorts and reflect underlying biological diversity within the PD spectrum [30].

Although none of the studies included in this review stratified patients by subtype, we propose that future investigations should examine whether IOS parameters vary according to PD progression phenotype. Such an approach could enhance the early detection of respiratory dysfunction, support individualized monitoring and align with precision medicine efforts in neurodegenerative disease management.

## 5. Conclusions

This systematic review provides emerging evidence that IOS may serve as a valuable adjunctive tool in the respiratory evaluation of patients with PD. Across the included studies, IOS parameters were shown to detect early and subtle alterations in respiratory mechanism, often in the absence of abnormalities on conventional spirometry. In addition, correlations between IOS values and clinical markers such as disease stage, spirometric indices and heart variability suggest that IOS may capture both motor-related and autonomic components of respiratory impairment in PD.

Importantly, the effort-independent nature of IOS makes it especially suitable for use in neurologically impaired populations who may have difficulty performing other test like spirometry. Future research should prioritize larger, longitudinal and multicenter studies to validate IOS as a diagnostic and prognostic tool in PD-related respiratory dysfunction. The standardization of testing protocols and integration with clinical staging and autonomic assessments would also strengthen its role in routine clinical practice. Until then, IOS should be regarded as a complementary method that offers unique physiological insights beyond those captured by traditional pulmonary function tests.

## Figures and Tables

**Table 1:** Summary of database search strategy.

Database	Search String Used	Articles Retrieved
PubMed	(“Parkinson” AND “oscillometry” AND “pulmonary function”) OR (“Parkinson” AND “impedance” AND “lung function”)	4
Web of Science		7
Scopus		4
ScienceDirect (Elsevier)		139
Google Scholar		47

**Table 2:** Characteristics and main findings of studies investigating impulse oscillometry in the assessment of respiratory function in Parkinson’s disease.

Authors	Year	Study Designs	Population	PD Stage	Technique	Main Findings
Caldas et al. [13]	2023	Observational, Cross-sectional	47 patients with PD, 20 controls	I–III H&Y	IOS, Spirometry, Manovacuometry	IOS detects early peripheral airway changes in PD; correlates with muscle weakness and has high diagnostic accuracy (AUC > 0.85)
Oliviera et al. [18]	2022	Observational, Cross-sectional	21 patients with PD, 20 controls	I–II H&Y	IOS, Spirometry	IOS values were within normal range in mild-stage PD; spirometry showed signs of incipient obstructive disorder; levodopa had minimal effect on pulmonary function
Sampath et al. [19]	2022	Observational, Cross-sectional	30 PD patients	I–II HY	IOS, Spirometry, HRV *	Increased R20 correlated with HRV indices, indicating autonomic dysfunction; IOS detected changes between disease stages
Sampath et al. [20]	2020	Observational, Cross-sectional	30 PD patients	I–II H&Y	IOS, Spirometry	R5 and R20 increased with disease severity; negative correlation with FEV1 and PEF, suggesting IOS is more sensitive than spirometry

* Abbreviation: HRV–heart rate variability.

**Table 3:** Comparative features of spirometry and IOS in neurological diseases, including Parkinson’s disease [14,15,17].

Feature	Spirometry	IOS
Measurement Type	Effort-dependent; requires maximal inspiration/expiration	Effort-independent; measured during tidal breathing
Main Parameters	FEV_1_, FVC, FEV_1_/FVC, PEFR	R5, R20, R5–R20, X5, AX, Fres
Assesses	Global ventilatory capacity, obstruction, restriction	Airway resistance and reactance (central and peripheral)
Sensitivity to Early Dysfunction	Limited, especially in early or subclinical stages	High; detects subtle peripheral airway impairment
Motor Requirement	High; affected by tremor, rigidity and fatigue	Low; suitable for patients with motor impairments
Use in Autonomic Assessment	Not applicable	Correlates with heart rate variability in PD
Interpretive Complexity	Widely familiar across clinical disciplines	Requires specialized understanding of impedance metrics

Legend: FVC = forced vital capacity; FEV_1_ = forced expiratory volume in 1 s; PEFR = peak expiratory flow rate. R5 = total airway resistance at 5 Hz; R20 = central airway resistance at 20 Hz; R5–R20 = frequency-dependent resistance (indicator of peripheral airway dysfunction); X5 = reactance at 5 Hz (lung compliance); AX = area under the reactance curve; Fres = resonant frequency.

**Table 4:** Clinical respiratory patterns in Parkinson’s disease: diagnostic correlations between spirometry and IOS [13,18,19,20].

Clinical Pattern in PD (Study)	Expected Spirometry Findings	Expected IOS Changes	IOS Superior?
Mild to moderate PD and smokers—Caldas et al., 2023 [13]	↓ FVC, ↓ PEF with disease progression	↑ Rp in early PD, ↑Fr in moderate PD; ↓ Cdyn	Yes—identifies progressive dysfunction and stratifies by stage
Mild PD (“on/off” Levodopa)—Oliveira et al., 2022 [18]	Incipient obstructive changes (↓ FEV_1_/FVC, ↓ PEF)	No significant IOS changes between groups	Partial—spirometry more sensitive in this context
Autonomic dysfunction (HRV correlation)—Sampath et al., 2022 [19]	Not reported in detail; focused on HRV	↑ R20 positively correlated with HRV indices; ↓ X5 in some cases	Yes—reveals autonomic modulation of airway resistance
Early vs. Mild PD (H&Y I vs. II)—Sampath et al., 2020 [20]	Often normal; no significant change across stages	↑ R5, ↑ R20 in stage II; R5 negatively correlated with FEV_1_, PEF	Yes—detects subclinical changes before spirometry

Abbreviation: Cdyn = dynamic compliance, reflects the lung’s elastic response during tidal breathing. ↓ decreased, ↑ increased.

## Data Availability

Data are contained within the article.
